# Multi-level predictors of sexual autonomy among married women in Nigeria

**DOI:** 10.1186/s12905-022-01699-w

**Published:** 2022-04-12

**Authors:** Bola Lukman Solanke, Olufemi Mayowa Adetutu, Kazeem Adebayo Sunmola, Ayodele Aderemi Opadere, Nurat Kehinde Adeyemi, Daniel Alabi Soladoye

**Affiliations:** 1 Department of Demography and Social Statistics, Obafemi Awolowo University, Ile-Ife, Nigeria; 2 Centre for Gender and Development Studies, Ekiti State University, Ado-Ekiti, Nigeria; 3 Department of Behavioral Studies, Redeemer’s University, Ede, Nigeria; 4 Department of Sociology, Kaduna State University, Kaduna, Nigeria

**Keywords:** Sexual autonomy, Sexual health, Reproductive health, Women, Nigeria

## Abstract

**Background:**

Extant studies have established diverse individual-level and relational-level predictors of sexual autonomy among women in different countries. However, information remains scanty about the predictors beyond the individual and relational levels particularly at the community level. This study examined the multi-level predictors of sexual autonomy in Nigeria. This was done to shed more light on the progression toward attaining women-controlled safe sex in Nigeria.

**Methods:**

This study adopted a cross-sectional design that utilised the 2018 Nigeria Demographic and Health Survey (NDHS) data. The study analysed responses from 8,558 women. The outcome variable was sexual autonomy, while the explanatory variables were individual-level (maternal age group, maternal education, nature of first marriage, parity, work status, religion, and media exposure), relational-level (spousal violence, type of marriage, spousal living arrangement, household wealth quintile, alcoholic consumption, family decision-making, and degree of marital control), and community-level characteristics (community residency type, geographic region, community literacy, female financial inclusion in community, female ownership of assets in community, and community rejection of wife-beating). Statistical analyses were performed using Stata version 14. The multilevel regression analysis was applied. Statistical significance was set at *p* < 0.05.

**Results:**

Findings showed that parity, nature of first marriage, maternal education, media exposure, work status, and religion were significant individual-level predictors, while spousal violence, degree of marital control, type of marriage, family decision-making, and household wealth quintile were significant relational-level predictors of sexual autonomy. Results further showed that community-level characteristics also significantly predicted sexual autonomy. The likelihood of sexual autonomy was lower among rural women (aOR = 0.433; 95% CI 0.358–0.524), while the odds of sexual autonomy were higher among Southern women (aOR = 3.169; 95% CI 2.594–3.871), women who live in high literate communities (aOR = 3.446; 95% CI 3.047–3.897), women who reside in communities with high female financial inclusion (aOR = 3.821; 95% CI 3.002–4.864), and among women who live in communities with high female ownership of assets (aOR = 1.907; 95% CI 1.562–2.327).

**Conclusion:**

Women’s sexual autonomy was predicted by factors operating beyond the individual and relational levels. Existing sexual health promotion strategies targeting individual and relational factors in the country should be modified to adequately incorporate community-level characteristics. This will enhance the prospect of women-controlled safe sex in Nigeria.

## Background

Women’s sexual autonomy refers to ability to refuse both risky and non-risky sexual relations, as well as the ability to request from partners the use of condom before intercourse whether in marital or non-marital relationships [1, 2]. Sexual autonomy is a human right [3, 4], an important indicator of women empowerment in the society [5], and a safe health behaviour that promotes the survival of neonates [2]. Evidence across many sub-Saharan Africa countries indicated that substantial proportions of women in marital unions lacked the power to refuse sex from partners. For instance, a Ghanian study observed that 18.6% of the women could not refuse sex from partners, while a recent Nigerian study reported that 41.0% of the women lacked power to refuse sex from partners [6, 7]. Evidence also showed that many women lacked the power to insist on partners’ use of condom before intercourse. This was evident in two quantitative studies where inability to ask partner to use condom ranged from 31.4% to 59%. Similarly, a high level of inability to ask partner to use condom was reported in a recent qualitative study [8–10]. This depicts unequal power relations among couples in many African countries [5, 11], where patriarchy continue to shape women’s sexual and reproductive behaviour [12, 13].

Diminished sexual autonomy among women also elevates the risks of adverse sexual and reproductive health outcomes such as sexually transmitted diseases [14, 15], unintended pregnancies [16, 17], unsafe abortions [18], and poor access to effective modern contraceptives [19, 20]. There is also evidence that lack of sexual autonomy is a key source of mental health challenge among childbearing women [21]. The Coronavirus pandemic represent additional challenge for women’s sexual autonomy due to increased demand for domestic responsibilities and care-giving at the home front [22, 23]. The continued total or partial lockdown of schools and places of work, and home isolation undermines women’s sexual liberty, which may be the reason for rising cases of spousal violence during the pandemic [24, 25]. Thus, it is imperative that more research should be conducted to improve understanding of the underlying predictors of sexual autonomy in different climes.

Extant studies in Nigeria have established varied individual-level and relational-level predictors of sexual autonomy among women in different parts of the country. These include knowledge of HIV transmission, employment, land ownership, number of living children, acceptance of wife-beating, household wealth quintile and place of residence [26], education [12], household decision-making [8, 27], and child marriage [7]. More predictors have been identified in other climes. In Bangladesh, intimate partner violence was found to hinder women’s reproductive autonomy [28], while an earlier study in the same country [29] revealed women’s rejection of wife-beating, region of residence, and knowledge of sexually transmitted infections as key predictors of women’s sexual and reproductive autonomy. In Kenya, a study reported that women who had undergone female genital mutilation had poor ability to refuse sex from their partners [10]. In Ethiopia, it was found that HIV awareness contributed substantially to women’s ability to request the use of condom during sexual relations [30].

In spite of these findings, information remains scanty about the predictors of sexual autonomy beyond the individual and relational levels particularly at the community level. This has inadequately accounted for the importance of community-level factors that interventions could target not only for promoting women’s sexual health, but also for reducing the burden of adverse reproductive health outcomes among women in the country. It is particularly important to identify community-level predictors in Nigeria because of the persistence of communal cultural beliefs, gender norms and practices [31, 32] that subjugate women’s sexual lives and general well-being to men’s control and authority. More often than not, the patriarchal system in Nigerian communities supports men’s dominance of power relations within households [33]. This makes it difficult for women to engage in negotiations for safer sexual relations or being involved in family reproductive decision-making [34]. Thus, community characteristics not only reinforce the independent effects on women’s sexual autonomy, but it may also signpost possible community-based strategies for promoting women’s sexual health in the country.

The study therefore examined the multi-level individual, relational, and community level factors that predict women’s sexual autonomy. The study was guided by the research question: what are the predictors of sexual autonomy at the individual, relational, and community levels? Findings will shed more light on the progression toward attaining women-controlled safe sex in Nigeria. It will also provide inputs for strengthening sexual health promotion as targeted in the current national health promotion policy [35]. The gender and power theory [36, 37] and the socio-ecological theory [38] provided the theoretical lenses of the study. On the one hand, the gender and power theory befit the Nigerian social structure because it has persistently remained a patriarchal system heavily tilted against women, and with several socio-cultural practices that continue to have adverse impact on women’s health, economic productivity and empowerment [31–34]. On the other hand, the socio-ecological theory asserts that social or health outcomes may be influenced by different factors that operate at different levels of the society [38].

## Methods

### Design and data source

This study adopted cross-sectional design that analysed secondary data from the 2018 Nigeria Demographic and Health Survey (NDHS). The 2018 NDHS was executed by the National Population Commission (NPC) in conjunction with the National Malaria Elimination Programme (NMEP). International development partners such as the United States Agency for International Development (USAID), Global Fund, Bill and Melinda Gates Foundation (BMGF), the United Nations Population Fund (UNFPA), and World Health Organisation (WHO) provided financial support for the implementation of the survey. Technical assistance for the survey was provided by ICF through the DHS Program being funded by the USAID [39]. The 2018 NDHS provides information for the estimation of basic demographic and health characteristics in the country.

### Sampling and participants

Comprehensive detail of the 2018 NDHS methodology has been published and widely available (https://dhsprogram.com/pubs/pdf/FR359/FR359.pdf). However, the basic methods are briefly described. The 2018 NDHS partitioned the country into two sampling strata, based on rural or urban residency, which yielded 74 sampling strata. Independent samples were then selected in every stratum through a two-stage procedure. In the first stage, enumeration areas were randomly selected, after which households were randomly selected in the enumeration areas. In the second stage, households were selected for the study. Eligible men and women were then randomly selected for interviews in the households. Eligible men and women in the households were selected using simple random sampling method. Interviews were completed for a total of 41,821 women and 13,311 men using DHS model questionnaires as the data collection tool. The study focused on the data generated on the female respondents from the survey. Out of the bulk of 41,821 women covered in the survey, the study analysed responses from 8,558 women. This excluded all women who were not currently married (12,105), those not included in the domestic violence module (20,981), and those less than age 15 (112). Women who reported traditional religion were also excluded (65) due to their small size which may pose difficulty for data analysis.

### Outcome variable

The outcome variable in the study was sexual autonomy. This was measured from responses to three different questions. One, women were asked if it is justified to refuse sex with a husband who is infected with a sexually transmitted disease. Two, women were asked if they could refuse sexual demand from their husbands. Three, women were asked if they could request their husbands to use condom before intercourse. Each question had a ‘Yes’ or ‘No’ response. Sexual autonomy was generated by combining the responses. Women who reported ‘Yes’ to the three questions were deemed to be ‘sexually autonomous’, and coded ‘1’, while other women were grouped as ‘not sexually autonomous’, and coded ‘0’. This measure is consistent with the measurement of sexual or reproductive autonomy in existing studies [2, 18, 29, 40, 41].

### Explanatory variables

**Fig. 1:**
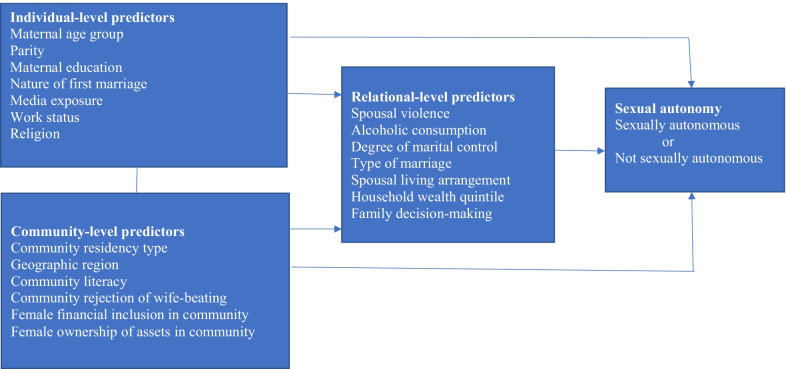
Conceptual framework based on socio-ecological theory and gender and power theory

Figure 1 presents the three sets of explanatory variables analysed. Firstly, seven individual-level characteristics already identified as important correlates of sexual and reproductive autonomy in existing studies [26, 40, 42–44] were selected. These are maternal age group (15–24, 25–34, and 35 +), maternal education level (none, primary, secondary, and higher), nature of first marriage (child marriage or not child marriage), parity (primiparity, multiparity, and grand multiparity), work status (employed or unemployed), religion (Islam or Christianity), and media exposure (low, moderate, and high). Media exposure was derived from the frequencies of reading newspaper, listening to radio, or watching television per week. Secondly, seven relational-level characteristics were selected based on existing knowledge that variables that characterised marital relationships are strong correlates of sexual and reproductive autonomy [6]. The variables selected are spousal violence (ever or never experienced at least one type of intimate partner violence), type of marriage (monogamy or polygyny), spousal living arrangement (living together or separately), household wealth quintile (poorest, poorer, middle, richer, and richest), alcoholic consumption (partner drinks or partner does not drink), family decision-making (male dominated or egalitarian), and degree of marital control (low, moderate, and high). Marital control was derived from husband’s controlling attitudes identified in existing studies as an important factor in promoting women’s autonomy and contraceptive behaviour [45, 46].

Thirdly, six community-level characteristics were selected. These are community residency type (rural or urban), geographic region (southern or northern), and community literacy (low, moderate, and high). Others are female financial inclusion in community (low, moderate, and high), which represents the proportion of women in the community who had bank accounts, female ownership of assets in community (low, moderate, and high), which represents the proportion of women in the community who owned land/house independently or jointly with husband. Community rejection of wife-beating (low, moderate, and high) was also included. This represents the proportion of women in the community who rejected wife-beating regardless of the reason. The selection of community characteristics was guided by the gender and power theory [36, 37], which depicts subjugation of women to the control and authority of men. The community characteristics were derived from women’s individual responses through aggregation of the responses at the primary sampling units.

### Data analysis

Statistical analyses were performed using Stata version 14 [47]. Sample characteristics including the prevalence of sexual autonomy were described using frequency distribution and percentages. All the explanatory variables were examined for multicollinearity using the Variance Inflation Factor (VIF). Based on literature [48, 49], any variable with a VIF score of 10 or more is suggestive of harmful collinearity, and should be eliminated from further analysis, though some researchers opined otherwise [50]. Result of the VIF indicated that none of the variable pose any challenge to further analysis. Bivariate analysis using unadjusted Odds Ratio (uOR) was then performed to purposely select variables into multivariable regression models with *p *value set at 0.025.

The multilevel regression analysis which consisted of mixed and random effects [51] was applied in the study because it is particularly suitable for the analysis of data with hierarchical structure [52] such as the multiple levels of influence investigated in the study. Three models were fitted excluding the empty model which examined the variations in sexual autonomy across the communities without the influence of the explanatory variables. Model 1 was fitted to estimate the influence of the individual-level characteristics. Model 2 controlled for the relational-level characteristics, while Model 3 was the full model which controlled for both the relational and community-level characteristics. The mixed-effects of the models were estimated using the adjusted Odds Ratio (aOR) with 95% confidence interval. The random-effects of the models were estimated using the Intra-Cluster Correlation Coefficient (ICC) to reveal the importance of the community-level characteristics as widely used in cluster surveys [53, 54]. The models were checked for adequacy using Akaike Information Criterion (AIC). This parameter is widely used in the selection of the model with the best goodness-of-fit from a collection of models [55]. The AIC values is expected to decline as more variables are introduced into the modelling. The model with the best goodness-of-fit informs the discussion of findings.

## Results

Table 1 presents the socio-demographic characteristics of respondents. As shown in the table, slightly more than two-fifths (41.8%) of the respondents were not sexually autonomous. Women in the age group of 25–34 years were dominant in the sample (43.4%). More than one-third of the women were either primiparous (37.7%) or grand multiparous (33.8%). Nearly half (47.1%) of the respondents got married as a child, though slightly more than half (52.9%) of the respondents got married at older ages. More than one-third of the women (37.1%) had no formal education. Among the respondents with educational attainments, secondary education was the most common educational level attained. Less than a quarter (23.2%) of the respondents had high media exposure, though, the proportion who had moderate media exposure was higher (46.0%). The majority (71.7%) of the respondents were employed. Moslem women were slightly more than Christian women in the sample (52.8% vs. 47.2%).

**Table 1:** Respondents’ socio-demographic characteristics

Characteristic	Number of women	Percentage	Characteristic	Number of women	Percentage
*Sexual autonomy*			*Type of marriage*		
Sexually autonomous	4979	58.2	Monogamy	6643	77.6
Not sexually autonomous	3579	41.8	Polygyny	1915	22.4
*Maternal age group*			*Family decision*		
15.24	1792	20.9	Egalitarian	3104	36.3
25–34	3709	43.4	Male dominated	5454	63.7
35 +	3057	35.7	*Spousal living arrangement*		
*Parity*			Living together	7555	88.3
Primiparity	3225	37.7	Living separately	1003	11.7
Multiparity	2438	28.5	*Household wealth quintile*		
Grand Multiparity	2895	33.8	Poorest	1538	18.0
*Nature of first marriage*			Poorer	1633	19.1
Child marriage	4031	47.1	Middle	1722	20.1
Not child marriage	4527	52.9	Richer	1784	20.8
*Maternal education*			Richest	1881	22.0
None	3179	37.1	*Community residency type*		
Primary	1383	16.2	Urban	3868	45.2
Secondary	3084	36.0	Rural	4690	54.8
Higher	912	10.7	*Geographic region*		
*Media exposure*			Northern	4801	56.1
Low	2634	30.8	Southern	3757	43.9
Moderate	3933	46.0	*Community literacy*		
High	1991	23.2	Low	3023	35.3
*Work status*			Middle	2729	31.9
Unemployed	2420	28.3	High	2806	32.8
Employed	6138	71.7	*Female financial inclusion in community*		
*Religion*			Low	4026	47.0
Christianity	4037	47.2	Middle	1590	18.6
Islam	4521	52.8	High	2942	34.4
*Spousal violence*			*Rejection of wife-beating in community*		
Ever experienced	2547	29.8	Low	3022	35.3
Never experienced	6011	70.2	Middle	1436	16.8
*Alcoholic consumption*			High	4100	47.9
Partner does not drink	6497	75.9	*Female ownership of assets in community*		
Partner drinks	2061	24.1	Low	3468	40.5
*Degree of marital control*			Middle	2831	33.1
Low	3577	41.8	High	2259	26.4
Moderate	3465	40.5			
High	1516	17.7			
Total	8558	100.0	Total	8558	100.0

*Source*: Authors’ analysis based on 2018 NDHS

The majority (70.2%) of respondents had never experienced any type of spousal violence. Likewise, the majority (75.9%) of respondents reported that their husbands does not drink alcohol. Almost equal proportions of the women experienced either low (41.8%) or moderate (40.5%) degree of marital control. The dominant type of marriage among the respondents was monogamy. Slightly more than one-third of the respondents (36.3%) reported egalitarian family decision-making, while the majority (63.7%) reported that family decision-making was male-dominated. Most of the respondents (88.3%) are living together with their spouses. Household wealth was similar among the respondents.

More than half of the respondents (54.8%) reside in rural areas compared to the 45.2% urban dwellers. More than half (56.1%) of the respondents were residing in the Northern region. Community literacy was low in more than one-third (35.3%) but nearly equal proportions of the women reside in communities with moderate or high literacy. Nearly half of the respondents (47.0%) live in communities with low female financial inclusion though a substantial proportion (34.4%) live in communities with high female financial inclusion. Nearly half of the women (47.9%) reside in communities with high communal rejection of wife-beating. More respondents (40.5%) compared to other women live in communities with low female ownership of assets. Result of the empty model (not shown) reveal that in the absence of the explanatory variables, the variations in sexual autonomy across the communities was substantial (ICC = 39.8%). Subsequent models fitted accounted for the importance of the different sets of the explanatory variables.

Table 2 presents results of the multilevel analyses. In Model 1, five individual-level characteristics, namely, parity, nature of first marriage, maternal education, media exposure, and religion significantly predicted the likelihood of sexual autonomy among the sampled women. However, the intra-cluster correlation coefficient (ICC = 32.2%) reveal that beyond the individual characteristics of the women, the context of the communities in which the women reside also makes significant contribution to the likelihood of sexual autonomy among them. The inclusion of the relational-level characteristics in Model 2 did not alter the predictive power of variables examined in Model 1. As shown in Model 2, the five individual-level characteristics remained significant predictors of sexual autonomy among the women. In addition, six relational-level characteristics, namely, spousal violence, degree of marital control, type of marriage, family decision-making, spousal living arrangement, and household wealth quintile significantly predicted sexual autonomy. Though, Model 2 affirmed that both the individual and relational level characteristics are strong predictors of sexual autonomy, evidence (ICC = 32.3%) was provided that the community contexts play important role in the likelihood of sexual autonomy among the women.

**Table 2:** Effects of individual, relational, and community characteristics on sexual autonomy

Characteristic predicting sexual autonomy	Model 1			Model 2			Model 3		
	AOR	*p* value	95% CI	AOR	*p* value	95% CI	AOR	*p* value	95% CI
*Maternal age group*									
15–24 ^RC^	1.000	–	–	1.000	–	–	1.000	–	–
25–34	0.923	0.331	0.784–1.085	0.925	0.354	0.784–1.091	0.887	0.322	0.700–1.124
35+	0.953	0.630	0.784–1.160	0.960	0.690	0.785–1.173	0.923	0.552	0.709–1.202
*Parity*									
Primiparity ^RC^	1.000	–	–	1.000	–	–	1.000	–	–
Multiparity	1.240*	0.003	1.073–1.433	1.221**	*p* < 0.001	1.112–1.341	1.215*	0.009	1.049–1.407
Grand multiparity	1.281*	0.004	1.081–1.518	1.481**	*p* < 0.001	1.306–1.679	1.286*	0.004	1.081–1.530
*Nature of first marriage*									
Child marriage	1.000	–	–	1.000	–	–	1.000	–	–
Not child marriage	1.205*	0.003	1.064–1.364	1.137*	0.047	1.002–1.290	1.267*	0.004	1.080–1.486
*Maternal education*									
None ^RC^	1.000	–	–	1.000	–	–	1.000	–	–
Primary	1.679**	*p* < 0.001	1.430–1.971	1.487**	*p* < 0.001	1.259–1.757	2.480**	*p* < 0.001	2.082–2.954
Secondary	2.920**	*p* < 0.001	2.478–3.442	2.377**	*p* < 0.001	1.992–2.837	5.284**	*p* < 0.001	4.524–6.171
Higher	4.235**	*p* < 0.001	3.301–5.432	2.879**	*p* < 0.001	2.195–3.774	7.905**	*p* < 0.001	6.219–10.049
*Media exposure*									
Low ^RC^	1.000	–	–	1.000	–	–	1.000	–	–
Moderate	1.340**	*p* < 0.001	1.179–1.524	1.249*	0.001	1.094–1.427	2.362**	*p* < 0.001	2.011–2.773
High	1.488**	*p* < 0.001	1.247–1.775	1.294*	0.007	1.074–1.559	4.245**	*p* < 0.001	3.367–5.354
*Work status*									
Unemployed ^RC^	1.000	–	–	1.000	–	–	1.000	–	–
Employed	1.004	0.949	0.886–1.137	0.954	0.472	0.839–1.085	1.754**	*p* < 0.001	1.539–1.999
*Religion*									
Christianity ^RC^	1.000	–	–	1.000	–	–	1.000	–	–
Islam	0.483**	*p* < 0.001	0.420–0.556	0.527**	*p* < 0.001	0.451–0.616	0.261**	*p* < 0.001	0.226–0.301
*Spousal violence*									
Ever experienced ^RC^				1.000	–	–	1.000	–	–
Never experienced				1.481**	*p* < 0.001	1.306–1.679	1.391	*p* < 0.001	1.272–1.521
*Alcoholic consumption*									
Partner does not drink ^RC^				1.000	–	–	1.000	–	–
Partner drinks				0.940	0.418	0.810–1.091	0.913	0.384	0.745–1.120
*Degree of marital control*									
Low ^RC^				1.000	–	–	1.000	–	–
Moderate				0.816*	0.001	0.722–0.923	0.782	*p* < 0.001	0.683–0.895
High				0.814*	0.013	0.693–0.958	0.743	0.001	0.626–0.882
*Type of marriage*									
Monogamy ^RC^				1.000	–	–	1.000	–	–
Polygyny				0.665**	*p* < 0.001	0.584–0.757	0.456**	*p* < 0.001	0.397–0.524
*Family decision*–*making*									
Egalitarian ^RC^				1.000	–	–	1.000	–	–
Male dominated				0.629**	*p* < 0.001	0.553–0.715	0.333**	*p* < 0.001	0.288–0.386
*Spousal living arrangement*									
Living together ^RC^				1.000	–	–	1.000	–	–
Living separately				0.666**	*p* < 0.001	0.566–0.783	0.584	0.073	0.325–1.051
*Household wealth quintile*									
Poorest ^RC^				1.000	–	–	1.000	–	–
Poorer				1.177	0.065	0.989–1.400	1.356*	0.002	1.116–1.647
Middle				1.476**	*p* < 0.001	1.223–1.781	2.809**	*p* < 0.001	2.338–3.375
Richer				1.447*	0.001	1.172–1.787	3.463**	*p* < 0.001	2.596–4.620
Richest				1.913**	*p* < 0.001	1.491–2.457	6.831**	*p* < 0.001	5.478–8.520
*Community residency type*									
Urban ^RC^							1.000	–	–
Rural							0.433**	*p* < 0.001	0.358–0.524
*Geographic region*									
Northern ^RC^							1.000	–	–
Southern							3.169**	*p* < 0.001	2.594–3.871
*Community literacy*									
Low ^RC^							1.000	–	–
Middle							2.327**	*p* < 0.001	1.940–2.792
High							3.446**	*p* < 0.001	3.047–3.897
*Female financial inclusion in community*									
Low ^RC^							1.000	–	–
Middle							2.854**	*p* < 0.001	2.367–3.441
High							3.821*	*p* < 0.001	3.002–4.864
*Rejection of wife*–*beating in community*									
Low ^RC^							1.000	–	–
Middle							1.149	0.335	0.866–1.524
High							1.492	0.088	0.942–2.361
*Female ownership of assets in community*									
Low ^RC^							1.000	–	–
Middle							1.401*	0.002	1.130–1.737
High							1.907**	*p* < 0.001	1.562–2.327
AIC	9963.27			9788.04			9753.99		
ICC	32.2%			32.3%			20.0%		

*RC* reference category

*p < 0.05, **p < 0.001

Model 3 ascertained the significance of the individual and relational level characteristics and provided further evidence of the role of the community characteristics (ICC = 20.0%). In the model, the likelihood of sexual autonomy was higher among multiparous women (aOR = 1.215; 95% CI 1.049–1.407) and grand multiparous women (aOR = 1.286; 95% CI 1.081–1.530) compared to women in the reference category. Women who married as adults were more likely to be sexually autonomous (aOR = 1.267; 95% CI 1.080–1.486) compared to those in early/child marriage. Women’s ability to be sexually autonomous improved consistently and significantly as women’s educational attainment also improves. Likewise, women who had moderate or high media exposure were more likely to be sexually autonomous compared to women who had low media exposure. Employed women were more likely to be sexually autonomous compared to unemployed women (aOR = 1.754; 95% CI 1.539–1.999). The odds of sexual autonomy were lower among Moslem women compared to Christian women (aOR = 0.261; 95% CI 0.226–0.301).

While women who had never experienced spousal violence had higher odds of sexual autonomy (aOR = 1.391; 95% CI 1.272–1.521), the odds were lower among polygynous women (aOR = 0.456; 95% CI 0.397–0.524), women experiencing either moderate (aOR = 0.782; 95% CI 0.683–0.895) or high marital control (aOR = 0.743; 95% CI 0.626–0.882), and women whose family decision-making were male-dominated (aOR = 0.333; 95% CI 0.288–0.386). The odds of sexual autonomy increased consistently as household wealth improved. Rural women were less likely to be sexually autonomous compared to urban women (aOR = 0.433; 95% CI 0.358–0.524). In contrast, Southern women were more than three times more likely to be sexually autonomous compared to Northern women (aOR = 3.169; 95% CI 2.594–3.871). The likelihood of being sexually autonomous increased progressively as literacy level, female financial inclusion, and female ownership of assets in the community improved from moderate to high levels.

## Discussion

This study was designed to identify factors predicting sexual autonomy beyond the individual and relational levels in Nigeria. This was an improvement upon existing studies [2, 19, 29, 40] that rarely made effort to identify community-level predictors of sexual autonomy. By identifying more predictive factors at the community level, the study not only accounts for the contribution of communal contexts to the state of women’s sexual and reproductive autonomy in Nigeria, but also provides support for the socio-ecological theory [38] by providing evidence of multilevel influences on sexual autonomy. Four key findings emerged from the study. One, the proportion of sexually autonomous married women in the country is 58.2% while another substantial proportion (41.8%) are not sexually autonomous. The proportion of sexually autonomous married women found in the study is higher than the 45.97% reported for Nigeria in a recent study [40]. The disparity in prevalence found in the two studies may be due to the differences in the target population of the studies. Based on the proportion who are not sexually autonomous, and considering that Nigeria is the most populous country in Africa, it is reasonable to infer that the absolute number of women who are not sexually autonomous in the country is high. This has serious implications for the demographic and health situation of the country.

On the one hand, it implies that large number of women in the country are vulnerable to unintended pregnancies and fertility [16, 17] which contribute to persistent high fertility in the country, and increase women’s childbearing and child rearing burden. Promoting women’s sexual autonomy may therefore be pivotal to women’s use of modern contraceptives. This view was substantiated in an earlier study in Nigeria [18], which found that the likelihood of contraceptive use was higher among sexually autonomous women. On the other hand, the incidences of sexually transmitted infections and unsafe abortion [14, 15, 18] may continue to increase in the country due to the inability of many women to control their sexual relations. This not only aggravate public expenditure on sexual and reproductive health in the country, but also calls for more attention on women’s sexual health in the country. This is particularly important in the current era of Covid-19 crisis. As evident worldwide [22, 23], the home care responsibilities of women increased greatly during the pandemic particularly in communities going through total or partial lockdown of institutions, and home isolation. In the midst of these growing caring responsibilities, unsolicited or unprotected sex may worsen the health of many women during the pandemic. Thus, both local and national efforts must be intensified to promote women’s sexual health, first by raising awareness that women’s sexual rights are human rights [3, 4] that must be respected and protected, and secondly by expanding strategies to capture more causal factors of sexual autonomy in the country.

Two, women’s individual characteristics are important factors that either deter or enhance sexual autonomy. As shown in the study, and in line with findings in existing studies, individual characteristics such as maternal education [26, 42], media exposure [40, 43], nature of first marriage [7, 40], and employment [40] play significant roles as levers of sexual and reproductive autonomy. This finding gives credence to many existing strategies on improving women’s characteristics particularly women’s education and economic status. As established in some studies [6, 41, 44], enhancing women’s education and economic productivity reduces the risks of child marriage, and increases women’s empowerment in terms of having adequate knowledge of reproductive health obstacles and improve access to needed reproductive health services. Exposure to mass media is also an important factor in improving women’s knowledge and prevention of sexually transmitted infections including HIV/AIDS. Bearing in mind that HIV/AIDS vaccine is yet to be developed for use, universal awareness of the causes and methods of preventing HIV/AIDS infection through the mass media appears to be the most potent tool for reducing further spread of the epidemic. This is because mass media outlets such as radio and television often reach large segments of people, which may be exploited purposely to influence perception, attitudes and behaviour. Thus, improving women’s social condition through education, information and empowerment foster health promotion initiatives particularly sexual and reproductive health interventions that seek to address the social conditions which serve as barrier to women’s sexual and reproductive health.

Three, relational factors such as spousal violence, family decision-making, marital control, polygyny, and household wealth significantly shape women’s sexual and reproductive autonomy. In most cases, if marital relationships are characterised by egalitarian practices, the health of women and children improves. On the other hand, if relationships are characterised by inequality and practices that tend to subjugate women under men’s control and authority, women’s health including sexual health becomes adversely affected. As shown in this study, and in agreement with existing studies [6, 8, 20, 26, 27, 41, 44], poor women’s sexual and reproductive health thrives amidst unequal power relations particularly in household or family decision-making. Evidence of inequality in unions abounds in the Nigerian social structure [31–34]. In the absence of legal and social support for behaviour change, married women who try to resist male dominance of unions mostly become victims of intimate partner violence. It is therefore imperative in Nigeria that women’s health promotion programmes should consider the development of strategies that seek to address conjugal issues that may have implications for women’s health. Though, the current policy [35] seeks to reduce structural and societal barriers to health services access and utilisation, however, no action has been taken to capture conjugal contexts. This could be achieved through the media strategy of the policy by developing behaviour change messages to be disseminated through social and mass media outlets.

Four, community features have a major impact on sexual autonomy. This is an essential factor that has been overlooked in many previous studies [2, 19, 29, 40]. This finding suggests that health authorities should take steps to mobilise community leaders to raise awareness about communal practices and norms that do not promote healthy sexual and reproductive life, particularly practices that do not recognise women as equal partners in marital relationships. It is well established in literature [9, 13, 31–34] in Nigeria and many developing countries that several cultural practices do not promote women’s health. While communities differ in terms of reproductive health beliefs, norms and practices, it is pertinent to note that sexual health promotion programmes that work effectively in one community may be ineffective in another community.

## Strengths and limitations of study

This study builds on earlier findings which identified the individual and household level predictors of women’s sexual autonomy but nearly ignored community-level predictors. This inadequately accounted for the significance of community-level factors in initiatives seeking to promote women’s sexual health in the country. This knowledge gap is now filled by findings in this study. A high-quality dataset was analysed in the study, which makes the study findings comparable to findings in studies across developing countries. The findings of this study are subject to the following drawbacks. One, the rule of 10 was applied in identifying multicollinear variables without further assessment of the VIF. Some authors have argued that further assessment should be made before eliminating variables from further analysis. Two, the data analysed in the study was cross-sectional, and therefore not sufficient to confirm prediction of sexual autonomy. Thus, the use of the term ‘predictor’ in the study simply connotes significant correlation of the research variables.

## Conclusion

This study examined the multi-level predictors of women’s sexual autonomy in Nigeria. Findings from secondary analysis of 2018 Nigeria Demographic and Health Survey datasets reveal that women’s sexual autonomy in the country was significantly predicted by multiple factors operating at the individual, relational, and community levels. Existing sexual health promotion strategies targeting individual and relational factors should be modified to adequately incorporate community-level characteristics. This will facilitate the attainment of women-controlled safe sex in the country. In addition, women’s health promotion programmes should consider the development of strategies that seek to address conjugal issues that may have implications for women’s health.

## Data Availability

Data analysed in the study is available in public domain and could be accessed online at https://dhsprogram.com/data/dataset/Nigeria_Standard-DHS_2018.cfm?flag=1.

## Abbreviations

AIC: Akaike Information Criterion
NDHS: Nigeria Demographic and Health Survey
NPC: National Population Commission
DHS: Demographic and Health Survey
WHO: World Health Organization
UNFPA: United Nations Fund for Population Activities
USAID: United States Agency for International Development
VIF: Variance Inflation Factor
ICC: Intra-Cluster Correlation Coefficient
